# Aloe vera derived carbon dots as multifunctional fluorescent probe for temperature, pH, and ferric ion sensing

**DOI:** 10.1038/s41598-025-34499-x

**Published:** 2026-01-20

**Authors:** A. Rezk, M. K. Elnimr, A. S. Atlam, M. M. Mosaad, M. Ghali

**Affiliations:** 1 Physics Department, Faculty of Science, Tanta University, Tanta, Egypt; 2 Physics Department, Faculty of Science, Kafrelsheikh University, Kafrelsheikh, Egypt; 3 Institute of Basic and Applied Sciences, Egypt-Japan University of Science and Technology, Alexandria, Egypt

**Keywords:** Aloe vera, Carbon dots, PH sensing, Powder-state sensor, Ferric ions sensing, Chemistry, Materials science, Nanoscience and technology, Optics and photonics

## Abstract

Carbon dots (CDs) synthesized from natural precursors with excellent fluorescent properties are drawing a lot of attention for sensing and biosensing applications. We offer a sustainable carbon precursor derived from *Aloe vera* gel, utilizing a green one-step hydrothermal synthesis of carbon dots. A systematic optimization of the synthesis parameters was conducted by varying the reaction temperature, ranging from 180 °C to 240 °C in a 20 °C interval, while adjusting the reaction duration (4, 8, 12, and 16 h). We studied the absorption and emission of the prepared CDs using optical characterizations, such as UV-Vis and photoluminescence spectroscopy. X-ray diffraction (XRD), transmission electron microscopy (TEM), and Fourier transform infrared (FTIR) spectroscopy were used to study the structure and shape of the dots. These tests confirmed the creation of uniformly dispersed, amorphous, and functionalized carbon dots. The quantum yield and time-resolved PL decay were used in the determination of the most optimal synthesis conditions, yielding the highest photoluminescence quantum yield (PLQY) at 240 °C and 12 h. Results show that the synthesis parameters have a clear impact on the quantum yield, absorption, photoluminescence (PL) spectra, and morphology. CDs synthesized at 240 °C for 12 h with a carbon-to-oxygen ratio of Rc/o ≈ 2.37 showed the highest PLQY and were selected as sensitive fluorescence probes. As a first CDs powder-state temperature probe, the PL-intensity decrease from 298 to 393 K evidence strong thermal responsivity over a wide range. The fluorescence works as a pH sensor by changing the emission-intensity ratio across pH 3–12 with two calibration lines. For metal-ion sensing, the CDs exhibit outstanding selectivity toward Fe³⁺ with near-complete PL quenching and a linear range 0–500 nM. Notably, the limit of detection (LOD) is 16.15 nM, the lowest among hydrothermally synthesized carbon dots (CDs) from natural precursors.

**Supplementary Information:**

The online version contains supplementary material available at 10.1038/s41598-025-34499-x.

## Introduction

Carbon dots (CDs) have attracted substantial interest in recent years owing to their distinctive photoluminescence (PL), chemical and electronic properties, biocompatibility, and low toxicity^1,2^. These characteristics position CDs as promising candidates to replace metal-based quantum dots in a wide range of applications, including bioimaging^3–5^, biosensing^6^, drug delivery^7,8^, light-emitting diodes and energy application^9,10^, and photocatalysis^11^. CDs can be synthesized via either top-down or bottom-up routes. Top-down strategies generally employ laser ablation^12^, arc discharge^13^, acidic oxidation^14^, or electrochemical methods^15^, whereas bottom-up approaches use molecular precursors that undergo thermal or combustion treatments^16^, microwave-assisted synthesis^17^, or other solution-based processes^18^. Among these techniques, hydrothermal synthesis is particularly attractive because it is simple, scalable, and environmentally benign. In this method, carbon precursors are dissolved in water and subjected to elevated temperature and pressure, inducing dehydration, carbonization, and nucleation to form CDs. Tuning parameters such as reaction temperature, time, and precursor concentration enables precise control over CD size, surface functionality, and PL behavior^19^. However, even though there have been a lot of studies on hydrothermally produced CDs, the link between controllable synthesis parameters (especially temperature and reaction time), surface-state evolution, and the resulting PL/lifetime response is still not systematically measured in many biomass-derived systems^9,20^. This gap makes it hard to design CDs for sensing applications, where even small changes in the functional groups on the surface can have great impacts on emission intensity, wavelength, and quenching behavior^21^. A variety of natural products, including milk^22^, green tea^23^, and banana^24^, contain abundant carbon-rich components such as cellulose, starch, and sugars that decompose hydrothermally to yield CDs, while phenolic compounds and lignin further enhance PL by contributing to the carbon dot structure. These natural precursors also provide functional groups such as hydroxyl (–OH), carboxyl (–COOH), amino (–NH₂), and carbonyl (–C = O), which play a key role in governing the surface chemistry and optical properties of CDs.​​ Among biomass precursors, *Aloe vera* gel is particularly attractive because its biopolymer-rich composition can generate fluorescence CD easily coordinate to metal-ions due oxygen-containing surface groups. *Aloe vera gel* is composed of approximately 98.5% water^25^. The remaining complex biopolymer mixture, 0.5–2% of the solid material, comprises various compounds, such as water-soluble and fat-soluble vitamins, minerals, enzymes, polysaccharides, phenolic compounds, and organic acids^26^. Therefore, *Aloe vera* gel provides an economical green precursor that can produce CDs with rich surface groups important for sensing applications.

Temperature is a fundamental physical parameter because it strongly influences chemical, physical, and biological processes. It can be monitored using sensors based on nanoparticles, rare-earth materials, and organometallic compounds^27,28^. However, only a limited number of studies have investigated CDs that exhibit temperature-dependent fluorescence^29,30^, and there is still a clear need for a simple and efficient synthetic route to CDs capable of operating over a broad temperature-detection range. Importantly, almost all existing reports emphasize performance (sensitivity/range) only depending on fluorescent nanothermometers in the solution phase. In parallel, the development of sensitive and reliable pH probes is essential for studying environmental phenomena and biological systems. CDs have emerged as attractive pH-sensing materials owing to their excellent biocompatibility, small particle size, and photostability, and CD-based sensors yield pH values in good agreement with those obtained from standard pH meters, validating their suitability for pH determination^31^.

Green-fluorescent CDs were solvothermally synthesized and utilized as a pH optical sensor where a linear correlation between the CDs photoluminescence (PL) intensity and pH (in the pH range of 6–9) was found^32,33^. On the other hand, CDs synthesized using a hydrothermal method were capable of detecting pH values across a broader range from 1 to 14^31^.

Ferric ions (Fe³⁺) are among the most widespread metal ions in the environment and the human body. High iron levels can cause serious health issues, such as kidney failure, liver damage, and even death^32^. Therefore, developing cost-effective analytical methods for identifying Fe^3+^ ions and other metal ions become crucial. Fluorescence quenching efficiency stands out among those methods as a particularly promising technique for heavy metal ion detection. However, fluorescence quenching can originate from multiple pathways (e.g., coordination-induced electron/energy transfer or optical inner-filter effects), so mechanistic interpretation benefits from combining steady-state PL with lifetime measurements and appropriate control experiments.

In this work, we examined the effect of the reaction temperature on the synthesis of CDs by conducting experiments at four different temperatures (180 °C, 200 °C, 220 °C, and 240 °C) for four different reaction times (4, 8, 12, and 16 h) each. We aimed to study the optical and structural properties and chemical composition of these CDs, using various techniques such as high-resolution transmission electron microscopy (HRTEM), X-ray diffraction (XRD), Fourier transform infrared (FTIR) spectroscopy, photoluminescence (PL), ultraviolet-visible (UV-vis) absorption, and PL lifetime measurements. By correlating FTIR-identified functional groups and time-resolved PL lifetimes with changes in PL emission/absorption features, we establish synthesis–structure–property relationships that support rational selection of conditions for sensing-relevant optical behavior.

## Experimental

### Raw materials and chemicals

Fresh  *Aloe Vera* was commercially obtained from local market in Alexandia, Egypt. Quinine sulfate and sodium hydroxide were purchased from Sigma Aldrich. Phosphoric acid, boric acid, and all metal ions Co(NO₃)₂·6 H₂O, CdCl₂·2 H₂O, MgSO_4_.7H_2_O, CaCl_2_, FeCl_3_, LiCl, NaCl, Ni(NO₃)₂·6 H₂O, AlCl₃·6 H₂O, ZnCl_2_, CuCl_2_.2H_2_O, HgCl_2_, FeCl_2_.4H_2_O were obtained from Fisher Scientific.

### CDs Synthesis

For the synthesis of CDs using a bottom-up method, a Teflon-lined autoclave (100 mL Teflon cup) was used to perform the hydrothermal treatment. The ratio of the volume of solution to the volume of the autoclave is around 0.6. The synthesis was performed at four separate hydrothermal temperatures, namely 180 °C, 200 °C, 220 °C, and 240 °C, for four different time intervals, from 4 to 16 h in 4 h increments. At each trial, the water-to-aloe vera ratio remained at a constant value of 10:3. The heating rate of the reactor was 10 °C min^-1^ until the set point was achieved. After heating, the autoclave was allowed to cool down to room temperature. The resulting solutions were then centrifuged at 5,000 rpm for 15 min to separate the CDs. Subsequently, the supernatant was filtered through a 0.22 μm membrane to remove large or agglomerated particles. The successful creation of CDs from *Aloe vera* gel was indicated by the appearance of a yellowish-brown aqueous solution that emitted an intense blue color under UV light. Afterwards, the obtained CD solution was vacuum freeze-dried to provide a powder for later characterization.

The PL of the obtained $\:{CD}_{240}^{12}$ (synthesized at 240 °C with reaction time of 12 h) was measured with a series of pH values at room temperature. Namely, the synthesized $\:{CD}_{240}^{12}$(v = 50 µL) with a concentration of 1.45 mg/mL were dispersed in different pH solutions (pH 3–12, v = 2 mL), which were prepared using a pH meter with sodium hydroxide and phosphoric, boric, and acetic acids. We collected the PL spectra under an excitation wavelength of 360 nm after rapid incubation.

### Characterization

Fluorescence spectral measurements were carried out using an F-2700 spectrofluorophotometer. Absorbance spectra were recorded using a dual-beam UV-visible spectrophotometer (U-3900). High-resolution transmission electron microscopy (HRTEM) images were acquired on a JEOL2100F microscope by drop casting an appropriate dilution of CD aqueous solution onto the carbon-coated copper grids. X-ray photoelectron spectroscopy (XPS) measurements were carried out by Thermo-Scientific K-Alpha X-ray photoelectron spectroscopy with a monochromatized Al Kα line source (200 W) and examined the surface properties. Zeta potential measurements were conducted using a Malvern Nano ZS zetasizer. X-ray diffraction (XRD) patterns of CDs were obtained using a Shimadzu XRD6100 with Cu-Kα radiation, λ = 1.5406 Å. Identification of functional groups was done using a Bruker Vertex 70 using KBR pellets. The fluorescence decay spectra were measured via nanosecond time-correlated single photon counting (Horiba 3000U). For the measurements of the temperature-dependent fluorescence spectra, CD powder spectra were recorded using a JASCO FP-8600 photoluminescence spectrofluorometer. The heater was coupled to the FP-8600. The spectra were collected during sample heating from 298 to 393 K, in steps of 5 K.

## Optical properties

**Fig. 1:**
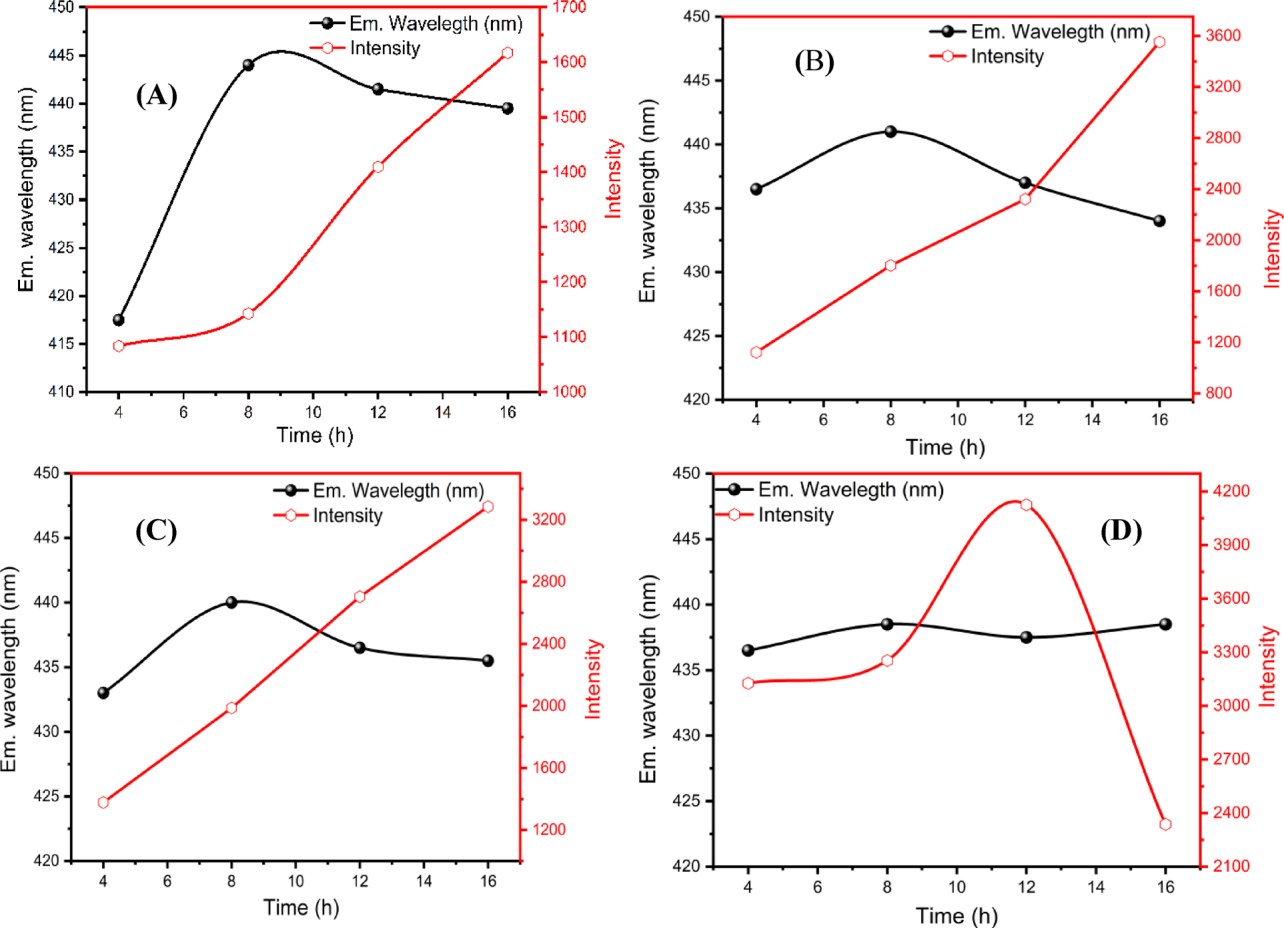
PL peak position wavelength of the synthesized CDs and their PL intensity versus the CDs synthesis reaction time at different synthesis temperatures; **A** 180 °C, **B** 200 °C, **C** 220 °C, and **D** 240 °C.

Initially, we extensively studied the effects of temperature and reaction time by synthesizing 16 samples. Each temperature was synthesized for four durations (4, 8, 12, and 16 h). In Fig. 1A, when heated at 180 °C for 4 h, small carbon nuclei form, often having a high density of oxygen-containing groups on the surface; these can lead to emissions at shorter wavelengths (blue-shifted). The PL is redshifted for longer synthesis time; this would be because of the formation of larger sp^2^ domains resulting from the high carbonization rate. For CDs synthesized at higher temperatures, see Figs. 1B–D. We observed only a slight wavelength shift instead of a large jump. This would be due to the equilibrium between the red shift in the PL wavelength due to generating more aromatic bonds and the loss or transformation of functional groups favoring the blue shift. Overall, PL intensity is enhanced due to the conversion of carbon precursor molecules in *Aloe vera* gel into carbonized cores with well-structured sp^2^ hybridization^43^. After reaching a peak intensity, the carbon dots can become overly carbonized. Such a rise-then-fall suggests there is an optimal condition at which maximum emission sites are formed. Beyond that, PL may degrade, causing the intensity to decrease. Overall, these trends suggest that the emission arises from multiple emissive centers governed by both sp²-domain evolution and surface/defect-related states.

**Fig. 2:**
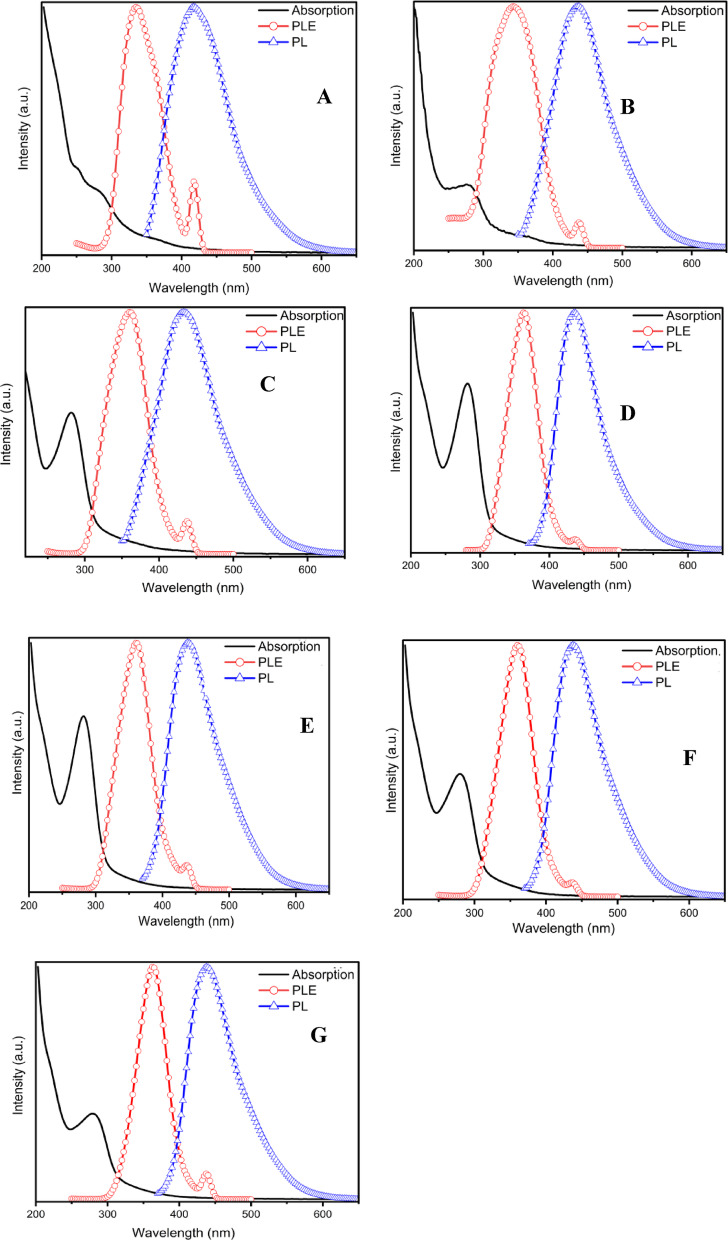
Normalized absorption, photoluminescence excitation (PLE), and photoluminescence (PL) of CDs samples synthesized at **A** 180 °C, **B** 200 °C, **C** 220 °C, **D** 240 °C for 4 h and **E**−**G** CDs samples synthesized at 240 °C for 8, 12 and 16 h, respectively.

Figure 2A shows the absorption spectra of the CDs; the sample synthesized at 180 °C exhibits three distinct spectral shoulders at 364 nm, 280 nm, and 250 nm. The shoulder at 364 nm is attributed to the n–π* transition of C = O bonds^54^kflows using a low-cost, sustainable precursor. Overall, this investigation a. The second shoulder, with an absorption maximum at 280 nm, is assigned to π–π* transitions of aromatic C = C bonds^34,35^. The shoulder at 250 nm is associated with transitions of C = C bonds in sp² carbon domains, and the aromatic π system contributes to the optical absorption peak of the CDs at 250 nm^36,37^. Figure 2B and C, and 2D present the absorption spectra of samples prepared at 200 °C, 220 °C, and 240 °C for 4 h, respectively. As the temperature increases, the absorption spectra reveal the disappearance of the absorption band at 364 nm, which may be attributed to a more uniform and less defective CD surface. In contrast, the absorbance at 280 nm in the shoulder region increases with increasing synthesis temperature, without any noticeable blue or red shift. When the temperature was raised from 200 °C to 220 °C, the absorption band at 280 nm increased by a factor of 3.7, and a further increase from 220 °C to 240 °C enhanced this band by a factor of 1.39. These observations indicate that the reaction temperature significantly influences the absorption properties of the as-synthesized CDs^38^.​ In Fig. 2E–G, the samples synthesized at 240 °C for 8, 12, and 16 h exhibit similar absorption peak types. However, increasing the reaction time leads to a decrease in absorption intensity, with almost no change in peak broadening. This reduction in absorption intensity is likely due to the removal of some surface functional groups accompanied by the restoration of C=C bonds, while the formation of additional sp²-hybridized carbon further extends the conjugated π system^39^. Photoluminescence excitation (PLE) provides information about the energy levels responsible for generating distinct emission bands. Figure 2 shows the normalized PLE and PL spectra of the CDs.​

The PLE spectrum corresponding to the strongest luminescence at an emission wavelength of 420 nm for the sample synthesized at 180 °C shows a PLE band at 335 nm. For the sample synthesized at 200 °C, the strongest luminescence at 435 nm corresponds to a PLE band at 340 nm. For the sample prepared at 220 °C, the strongest luminescence at 440 nm corresponds to a PLE band at 360 nm. Finally, for the sample obtained at 240 °C, the strongest luminescence at 440 nm corresponds to a PLE band at 362 nm. Time-dependent carbon dots were also synthesized at 240 °C for 8, 12, and 16 h; these CDs exhibit the strongest luminescence at 438 nm, which corresponds to the same PLE band at 360 nm.

**Fig. 3:**
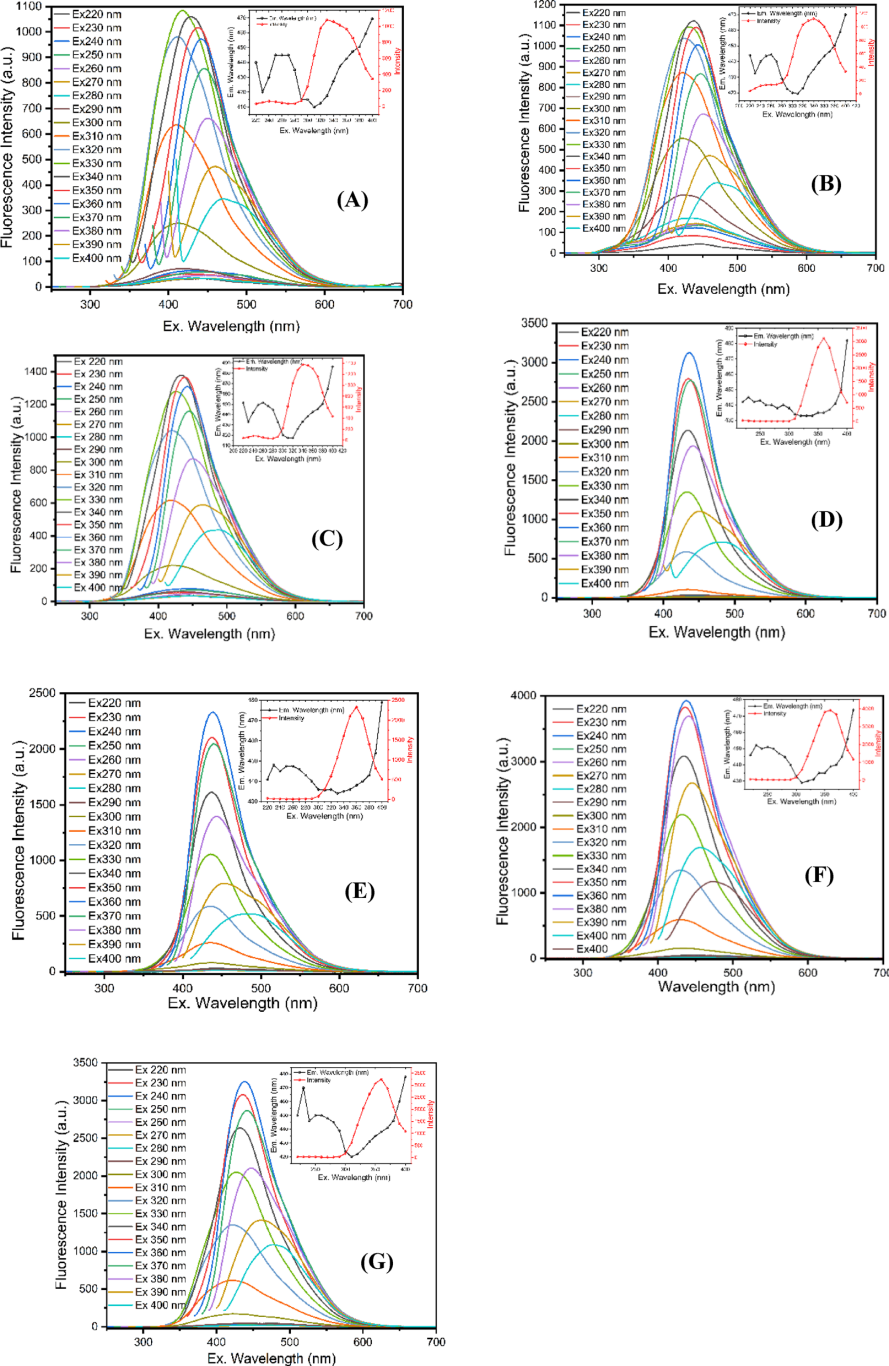
The photoluminescence spectra were obtained for the solutions of CDs using excitation wavelengths spanning from 220 nm to 400 nm, with increments of 10 nm of samples synthesized at **A** 180 °C, **B** 200 °C, **C** 220 °C, **D** 240 °C for 4 h and **E**−**G** synthesized at 240 °C for 8, 12 and 16 h, respectively. The inset figure displays the relationship between the maximum emission and the excitation wavelengths.

To obtain a more in-depth understanding of the optical characteristics, a fluorescence study was carried out using different excitation wavelengths. The PL spectra of the CDs were examined, and, as shown in Fig. 3, the CDs exhibit fluorescence with nearly symmetrical emission peaks. The PL intensity increases as the excitation wavelength is raised. In Fig. 3A, the PL intensity begins to increase at an excitation wavelength of 290 nm and reaches its maximum at 330 nm. Further increases in the excitation wavelength from 340 to 400 nm led to a decrease in emission intensity. When the excitation wavelength is 220 nm, the blue emission band starts at approximately 440 nm, shifts down to 410 nm as the excitation is increased to 310 nm, and then shifts up to around 470 nm at an excitation of 400 nm. An off-trend shift is observed around the main absorption band at 310 nm. In contrast to the behavior of all emission wavelengths, when exciting within the range of the absorption shoulders, the blue emission exhibits a pronounced shift from 450 to 440 nm down to 424–410 nm. Overall, the emission peak shows a progressive redshift from 415 to 470 nm as the excitation wavelength increases from 300 to 400 nm.

In Fig. 3B and C, the emission peak intensity for the CDs in B begins to increase at an excitation wavelength of 280 nm, whereas for the CDs in C, this increase starts at 290 nm. Both samples reach their maximum emission intensity at an excitation wavelength of 340 nm, with emission peaks at 435 nm for the CDs in B and approximately 433 nm for the CDs in C. For the blue emission peak of the CDs in B, the initial emission wavelength is 444 nm, which shifts to around 420 nm as the excitation wavelength increases to 310 nm. Beyond this point, further increases in excitation wavelength induce a red shift, with the emission moving from 420 to 470 nm. Similarly, for the CDs in C, the blue emission peak initially appears at 452 nm and undergoes a blue shift to 418 nm at an excitation wavelength of 320 nm, followed by a red shift to 487 nm as the excitation wavelength is further increased.

For CDs in D and E, the emission intensity starts increasing at excitation wavelengths of 300 nm and 290 nm, respectively. The maximum emission intensity for both sets of CDs is reached at an excitation of 360 nm. In terms of the blue peak, CDs in D initially emit at 442 nm, which undergoes a blue shift to 433 nm at 340 nm excitation before experiencing a redshift, extending the emission to 482 nm with further increases in excitation wavelength. In the case of CDs in E, the blue peak starts at 450 nm, shifting to 420 nm under an excitation of 310 nm, and then red shifts to 478 nm as the excitation wavelength increases.

In Fig. 3F and G, the emission intensity of the CDs in F begins to increase at an excitation wavelength of 300 nm and reaches a maximum at 360 nm. The CDs in G exhibit similar behavior, with the intensity starting to increase at 290 nm and peaking at 360 nm. Regarding the blue emission peak, the CDs in F initially emit at 446 nm; as the excitation wavelength increases to 310 nm, the emission shifts to 429 nm, followed by a redshift to 474 nm. For the CDs in G, the blue emission starts at 441 nm and shifts to 434 nm at an excitation wavelength of 330 nm, before undergoing a red shift to 479 nm. The photoluminescence of the CDs is clearly influenced by the excitation wavelength: beyond the excitation wavelength that yields the maximum intensity, any further increase leads to a gradual decrease in emission intensity. Origin of PL in carbon dots is typically explained by multiple emissive centers. Excitation-dependent PL is commonly associated with a distribution of emissive sites, including surface/edge trap states created by oxygen-containing groups and structural defects, as well as non-uniform sp² carbon domains; under different excitation wavelengths, different subsets of states can dominate, producing excitation-dependent emission^16,40–42^. In graphene-oxide/graphene-quantum-dot systems, PL has been attributed to isolated sp² clusters embedded in an sp³ (oxidized) carbon matrix, where improved uniformity and passivation reduce excitation dependence^43^. In bottom-up carbon-dot syntheses, fluorophore-like conjugated units formed during carbonization (e.g., pyridine-derivative motifs) can dominate the blue band, while defect states contribute additional longer-wavelength channels^44^. Therefore, biomass-derived CDs may show a combined contribution from sp²-domain emission, surface/defect traps, and possible molecular-state emitters, depending on synthesis conditions and purification.

**Fig. 4:**
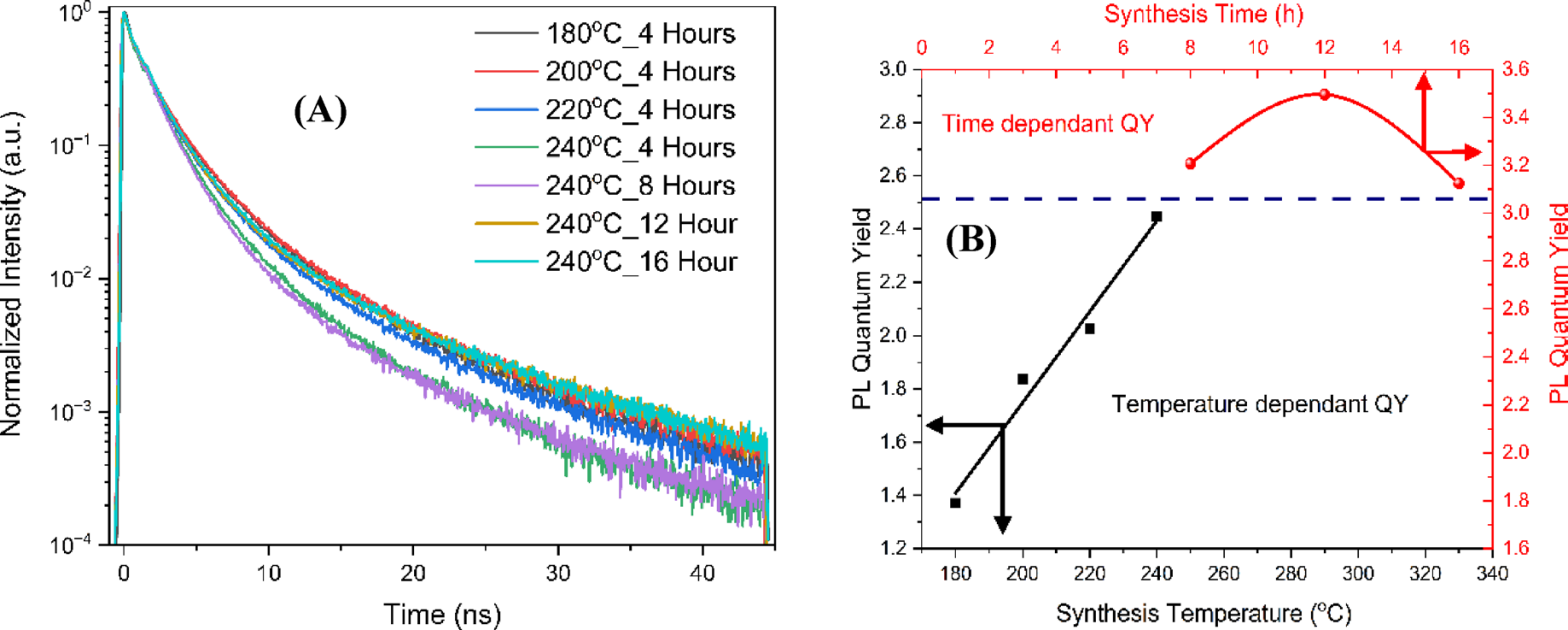
Time-resolved PL decay curves (**A**) and PL quantum yield (**B**) for samples synthesized at temperatures of 180 °C, 200 °C, 220 °C, and 240 °C with a fixed reaction time of 4 h, and at reaction times of 8 h, 12 h, and 16 h at a constant temperature of 240 °C.

For more study of the optical properties of the CDs (Fig. 4A), the PL time-resolved lifetime measurements were done by using time-correlated single photon counting. The decay data was fitted using the biexponential function Y(t), given by Eq. (1):

1 $${\mathrm{Y}}\left( {\mathrm{t}} \right){\text{ }} = {\text{ A}}_{{\mathrm{1}}} *{\mathrm{exp}}( - {\mathrm{x}}/\tau _{{\mathrm{1}}} ){\text{ }} + {\text{ A}}_{{\mathrm{2}}} *{\mathrm{exp}}( - {\mathrm{x}}/\tau _{{\mathrm{2}}} )$$

Where A_1_ and A_2_ are fractional contributions of the PL decay lifetimes τ_1_ and τ_2_, the average lifetime τ_average_ calculation was calculated using Eq. (2):

2 $$\:{\tau\:}_{average}=\raisebox{1ex}{${A}_{1}{\tau\:}_{1}^{2}+{A}_{2}{\tau\:}_{2}^{2}$}\!\left/\:\!\raisebox{-1ex}{${A}_{1}{\tau\:}_{1}+{A}_{2}{\tau\:}_{2}$}\right.$$

From the biexponential decay components, both intrinsic and surface defect states contribute to electron-hole recombination in the CDs^45^. Typically, emissions from surface defect states exhibit longer recombination lifetimes compared to those from intrinsic states^42,46^.

Table 1 shows the fitted values for the lifetimes (τ_1_ and τ_2_) and the calculated average lifetime (τ average). Our lifetime calculations show that increasing the synthesis temperature of CDs in the range from 180 °C to 240 °C with an interval of 20 °C for 4 h reduces their photoluminescence lifetime because of delocalized π-electrons and decomposition of surface function groups. These factors lead to a shorter photoluminescence lifetime by creating non-radiative recombination pathways. However, extending the synthesis time at 240 °C temperature for 8, 12, and 16 h increases the lifetime. This increase in synthesis time would help in defect healing, the formation of stable surface functional groups, and a high degree of crystallization, resulting in fewer non-radiative decay pathways.

**Table 1:** The values of τ_1_, τ_2,_ and τ_average_ lifetime of the seven samples under the excitation λ_exc_ = 372 nm.

Sample		τ_1_ (ns)	τ_2_ (ns)	τ _average_ (ns)
Temperature (°C)	Time (Hour)			
180 °C	4 h	1.86	4.26	2.24
200 °C	4 h	1.87	4.65	2.26
220 °C	4 h	1.74	4.96	2.12
240 °C	4 h	1.57	4.16	2.16
240 °C	8 h	1.53	4.80	2.48
240 °C	12 h	1.56	4.57	2.66
240 °C	16 h	1.70	5.30	2.92

Adjusting the synthesis temperature slightly improves the lifetime of CDs by reducing surface defects, which can trap electrons or holes, or by introducing functional groups that stabilize the surface, thereby lowering the likelihood of defects forming. However, while the samples synthesized at 240 °C show fluctuating lifetimes over 4, 8, 12, and 16 h, their lifetimes are still consistently higher than any value obtained from samples synthesized at lower temperatures. This suggests that, despite some variability, higher synthesis temperatures contribute to longer recombination lifetimes due to the reduction in surface defects.

We calculated the PL quantum yield with the following equation:

$$\:{QY}_{s}={Q}_{ref}\times\:\left[\frac{{M}_{s}}{{M}_{ref}}\right]\times\:\left[\frac{{n}_{s}^{2}}{{n}_{ref}^{2}}\right]$$

In this equation, the subscript *S* represents synthesized CDs, and the subscript *ref* represents the standard reference (Quinine Sulfate with a 54% QY prepared in 0.1 mol/L sulfuric acid solution), *M* is the slope from the plot of the integrated PL area against the absorbance of the CDs or the Quinine Sulfate at various concentrations, and *n* is the refractive index of the solvent.

It was observed that the PLQY (Fig. 4B) depends on both the synthesis temperature and reaction time. At 180 °C, the PLQY is 1.37. As the synthesis temperature increases, the PLQY correspondingly rises, which can be attributed to enhanced radiative electron–hole recombination efficiency due to improved crystallinity, a reduction in defects, or more effective surface passivation. When the synthesis temperature reaches 240 °C, the PLQY continues to increase, and a maximum value of 3.5 is obtained at 240 °C after 12 h, followed by a decrease at 16 h of synthesis. The formation of defects through overgrowth or aggregation can promote nonradiative recombination pathways, thereby reducing the PLQY. In addition, several studies have shown that oxygen-containing functional groups, acting as nonradiative recombination centers, can suppress the intrinsic state emission associated with the sp² subdomains, resulting in a decreased PLQY^47,48^.

**Fig. 5:**
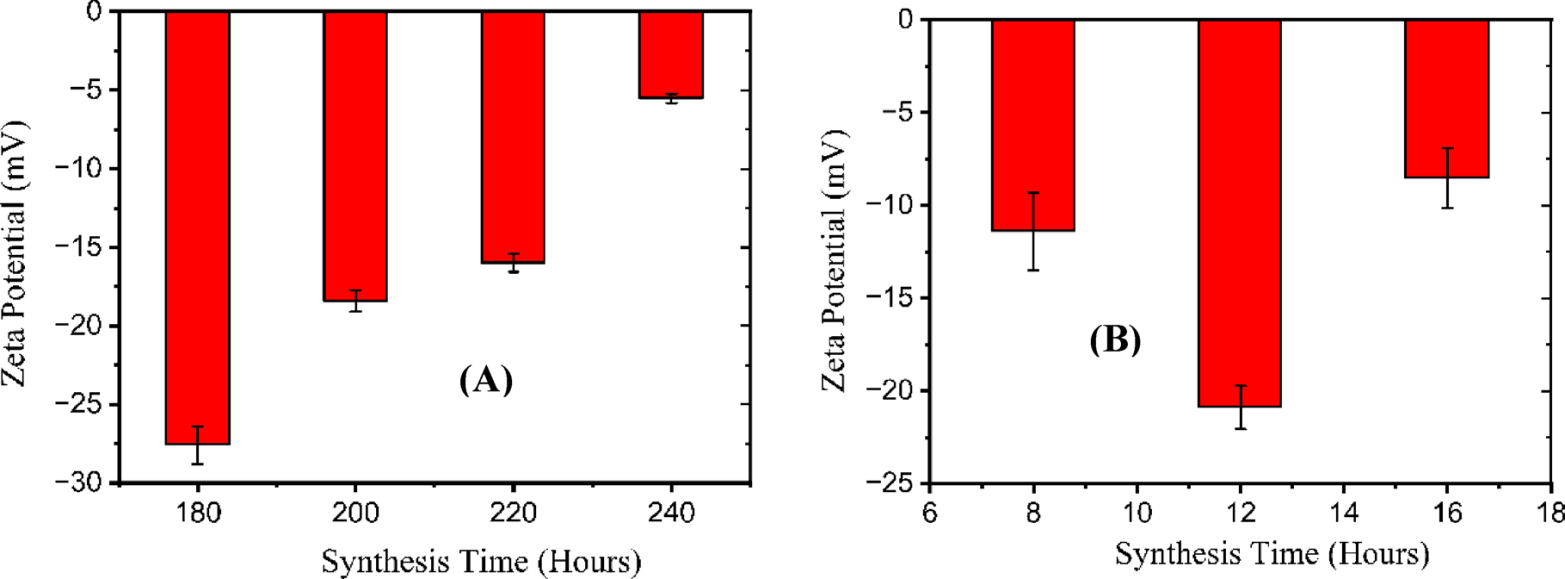
**A** Zeta potential values for CDs synthesized at temperatures of 180 °C, 200 °C, 220 °C, and 240 °C with a fixed reaction time of 4 h, **B** Zeta potential values for CDs synthesized at reaction times of 8, 12, and 16 h at a constant temperature of 240 °C.

The zeta potential was measured for samples prepared at 180 °C, 200 °C, 220 °C, and 240 °C for 4 h, as shown in Fig. 5A. The measurements show that all CD samples possess negative surface charges due to the presence of oxygen-containing functional groups^49^, with values of − 27.6 mV, − 18.4 mV, − 16.0 mV, and − 5.51 mV, respectively. The magnitude of the negative zeta potential decreases as the synthesis temperature increases. This trend suggests that higher synthesis temperatures lead to CDs with denser carbon cores, arising from the formation of more aromatic structures but a reduced number of surface functional groups^50^.

In Fig. 5B. We also presented the zeta potential for samples at 240 °C for durations of 8, 12, and 16 h. The values were also across all samples: −11.4 mV for the 8 h, −20.9 mV for the 12 h, and − 8.51 mV for the 16 h. The CDs at 12 h exhibited the highest magnitude of negative zeta potential, indicating that the reaction had reached completion. The lower negative zeta potential seen in the 16-hour sample could be because of overgrowth or aggregation, which lowers net surface charge.

**Fig. 6:**
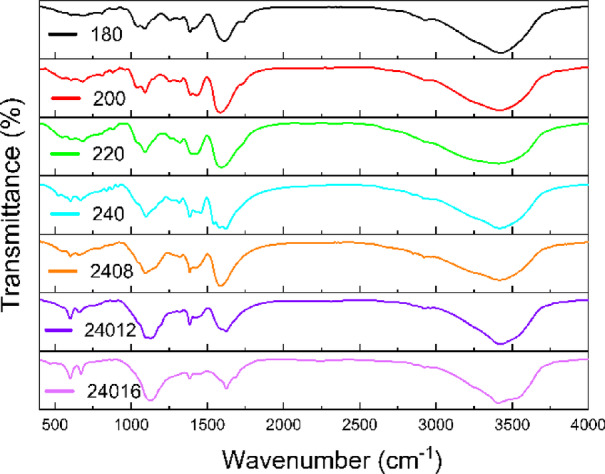
FTIR spectra of CDs samples synthesized at 180 °C, 200 °C, 220 °C, 240 °C for 4 h and samples synthesized at 240 °C for 8, 12 and 16 h, respectively.

In Fig. 6, the Fourier transform infrared spectroscopy (FTIR) analysis was conducted on powdered CDs to investigate the effect of time and temperature on the various functional groups and chemical bonds linked to the CDs. The wavenumbers at the broadening peak at 3420 cm^–1^ are typically associated with O–H stretching vibrations, indicating the presence of hydroxyl groups (–OH). The wavenumber 2919 cm^–1^ is associated with the C–H stretching vibrations in aliphatic compounds. Strong intensity peaks at 1607 cm^–1^ and 1579 cm^–1^ are attributed to the C=C stretching vibrations in aromatic rings. It is a characteristic band for the stretching mode of carbon-carbon double bonds. The peaks around 1424 cm^–1^ and 1388 cm^–1^ are attributed to the vibrations of methylene (–CH₂–) and methyl (–CH₃) groups, respectively. These peaks would be indicative of aromatic ring vibrations, specifically in-plane C-H bending vibrations or C-C stretching vibrations within the aromatic ring. Additionally, the wavenumber 1087 cm^–1^ is commonly associated with C-O stretching vibrations. It can be indicative of oxygen-containing functional groups such as hydroxyl (-OH), epoxy (C-O-C), or carboxylate groups (COO⁻). Notably, once the carbonization level is achieved, the synthesis no longer significantly degrades the surface functional groups, resulting in CDs with a uniform surface chemistry.

## Morphology of the cds

**Fig. 7:**
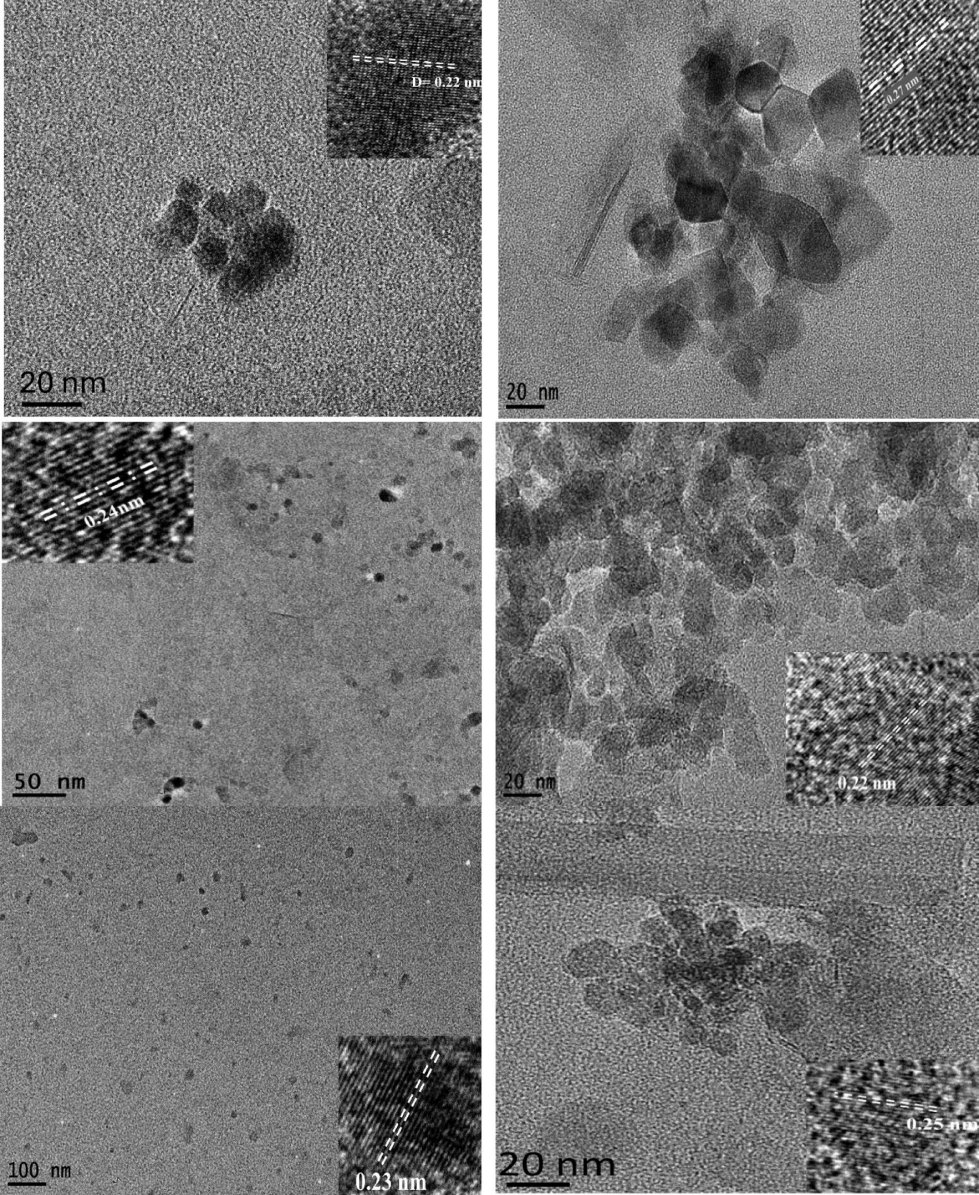
TEM images of the CDs, illustrating morphological changes in the morphology and size associated with changing in temperature and time of synthesis. **A** 180 °C, **B** 200 °C, **C** 240 °C, for 4 h and **D**−**F** synthesized at 240 °C for 8, 12 and 16 h, respectively.

Temperature and time had a direct effect on the average diameter of the obtained CDs. Flake-like structures can be seen in CD_180_ with sizes of approximately 430 nm. In addition to the flakes, there are smaller spherical CDs with sizes around 10 nm (Fig. 7A). CD_200_ (Fig. 7B) exhibited an obvious stripe-like hexagonal structure and was detected with an average size of 23.6 nm. For CD_240_ (Fig. 7, C), the average was ≈ 9 nm, with a spherical structure clearly seen. These results suggest that by increasing the temperature, the size decreases through accelerating carbonization, leading to the formation of more condensed carbon structures. Also, the lattice structure of the CDs was changed from a strip-like hexagonal to a spherical shape. For the time-dependent samples CD_8_, CD_12_ and CD_16_ (Fig. 7D and E), the average sizes were approximately 13, 16.6, and 9.3 nm, respectively. By increasing the reaction time, CDs nuclei continue to grow through further polymerization, aromatization, and carbonization, resulting in larger CDs. However, at 16 h (Fig. 7F), the CDs may undergo over-carbonization, leading to excessive cross-linking^39^ and structural strain, which induces new sp^2^-hybridized carbon and either enlarges existing sp² carbon nano islands or creates smaller sp² carbon configurations, resulting in a size reduction to ~ 10 nm. The interplanar spacing (d-spacing), shown in the inset, was determined from the selected lattice region by applying the inverse fast Fourier transform (IFFT). Based on the IFFT-derived lattice fringes, the measured d-spacings were 0.22 nm for CDs synthesized at 180 °C, 0.27 nm at 200 °C, 0.24 nm at 240 °C, 0.22 nm at 240 °C for 8 h, 0.23 nm at 240 °C for 12 h, and 0.25 nm at 240 °C for 16 h. These variations in d-spacing with synthesis temperature and duration suggest that the reaction conditions modulate the short-range structural ordering.

**Fig. 8:**
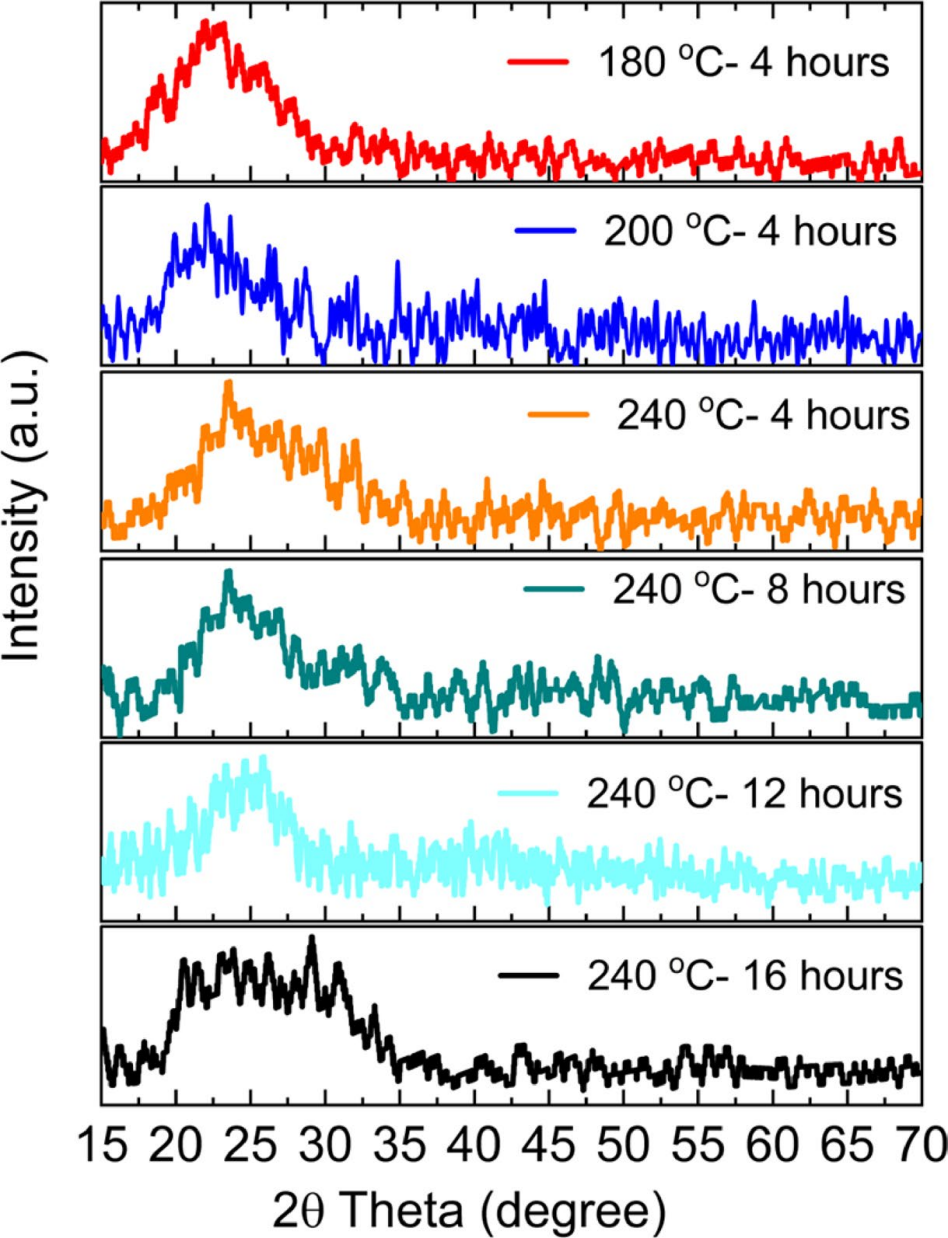
The XRD pattern of carbon dots.

The XRD diffraction patterns of the CD samples are shown in Fig. 8. Samples synthesized at 180 °C, 200 °C, and 220 °C for 4 h exhibit broad diffraction peaks centered at 2θ = ∼22°, indicative of amorphous or less ordered carbon^51^. In contrast, samples synthesized at 240 °C for 4, 8, 12, and 16 h show peaks at 2θ = ∼24°^52^. This shifts to higher diffraction angles because of the decrease in interlayer spacing^53^ and removal of some functional groups at the CDs surface, which corresponds to more crystalline graphitic structures as a function of both temperature and time.

**Fig. 9:**
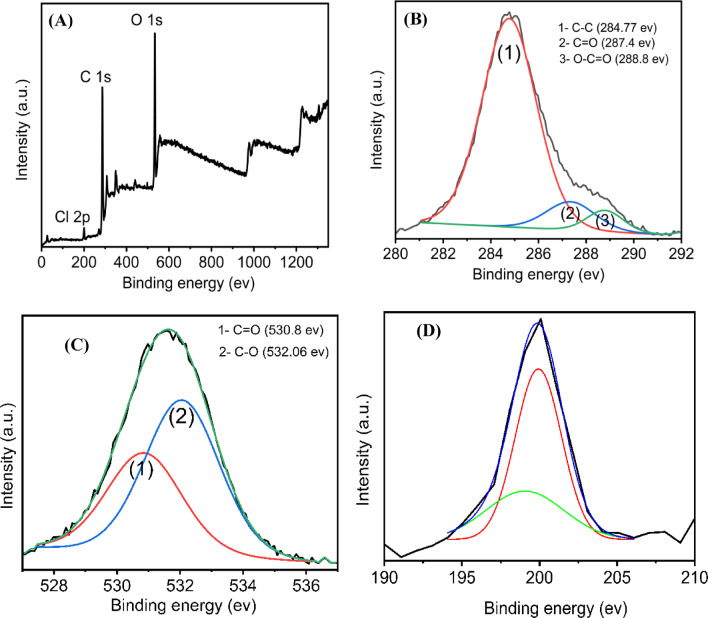
**A** Full-scan XPS profiles of CDs, **B**−**D** high-resolution spectra for C 1 s, O 1 s, and Cl 2p with peak deconvolution.

This made it possible to comprehend in great depth how temperature affects surface functional groups, particle size, and optical characteristics. The $\:{\mathbf{C}\mathbf{D}}_{240}^{12}$ show effective functional groups, uniform dispersion, and strong fluorescence emission intensity compared to other prepared samples, was further investigated for applications. In Fig. 9, the $\:{\mathbf{C}\mathbf{D}}_{240}^{12}$ composition was studied with X-ray photoelectron spectroscopy (XPS) and resulted in 68.57% carbon, 28.89% oxygen, and 2.4% chlorine, chlorine ions naturally present as *Aloe vera gel* composition based on water. In Fig. 9A, two peaks appear at 533.7 eV and 287.4 eV, attributed to O1s and C1s, respectively. The C1s can be deconvoluted into three different peaks centered at 284.77, 287.4, and 288.8 eV, which correspond to C–C, C = O, and O–C = O, respectively (in Fig. 9B). The O1s deconvolution reveals two bonding states with the presence of C = O and C − O components at 530.8 and 532.06 eV, respectively (in Fig. 9C). The Cl 2p XPS spectrum in Fig. 9D displays double peaks at 198 and 199 eV, which could be assigned to the binding energies of Cl 2p_3/2_ and Cl 2p_1/2_, respectively. This results further confirm the existence of carboxyl/hydroxyl groups in both CDs. Synthesis at this condition made the carbon-to-oxygen atomic ratio (R_c/o_) to be around 2.37.

**Fig. 10:**
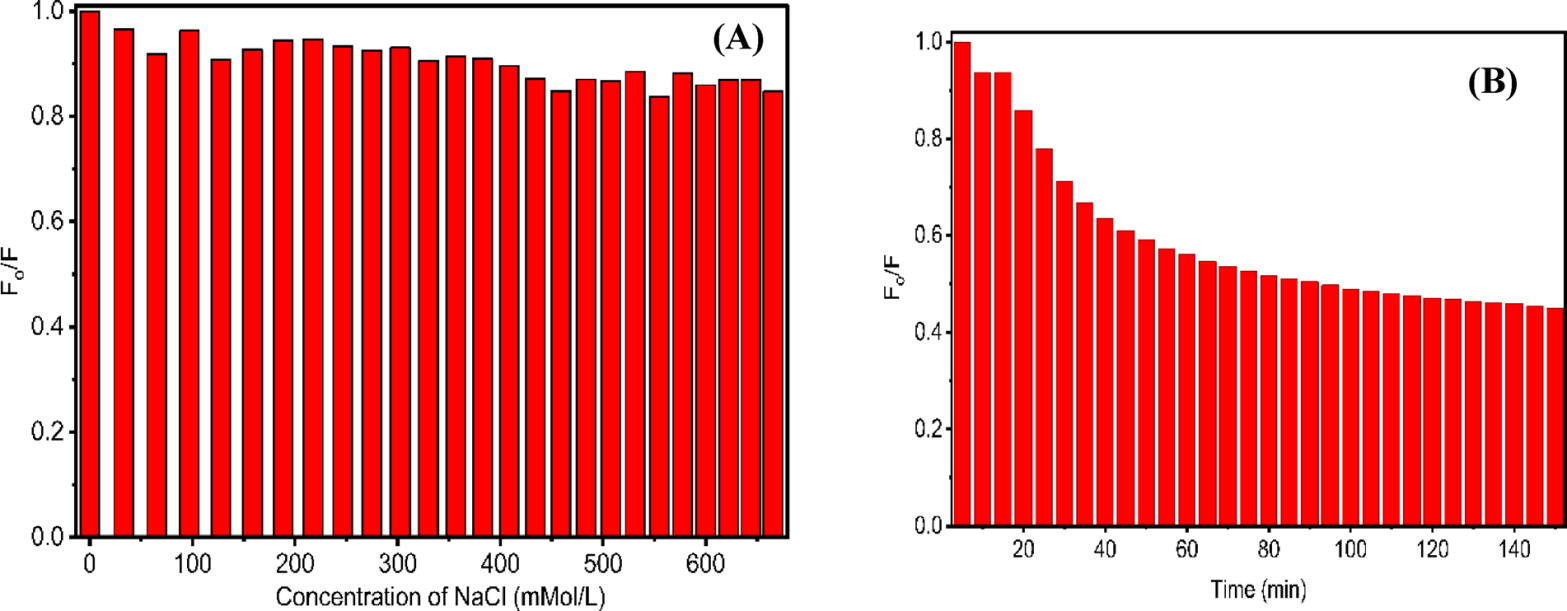
**A** Fluorescence Intensity of CD in aqueous NaCl solutions from 0 to 666 mM, **B** Photostability of the CDs under continuous 360 nm illumination for 150 min.

The stability of$\:{\:\boldsymbol{C}\boldsymbol{D}\boldsymbol{s}}_{240}^{12}$ carbon dots in the presence of different concentrations of NaCl was examined, revealing that fluorescence intensity remains nearly constant from 0 to 666 mM concentrations, Fig. 10A. In addition, the photostability test was examined under continuous excitation at 360 nm for 150 min with a 5-minute step, using a spectrofluorometer equipped with a xenon lamp (150 W). As shown in Fig. 10B, the fluorescence intensity of the **CDs** decreased slowly to ≈ 66% of their initial fluorescence intensity: this degradation may be caused by chemical bond cleavage due to oxidation. However, the fluorescence intensity at ≈ 440 nm decreased with light exposure; there wasn’t any peak shift. (Fig. 1 Supplementary).

**Fig. 11:**
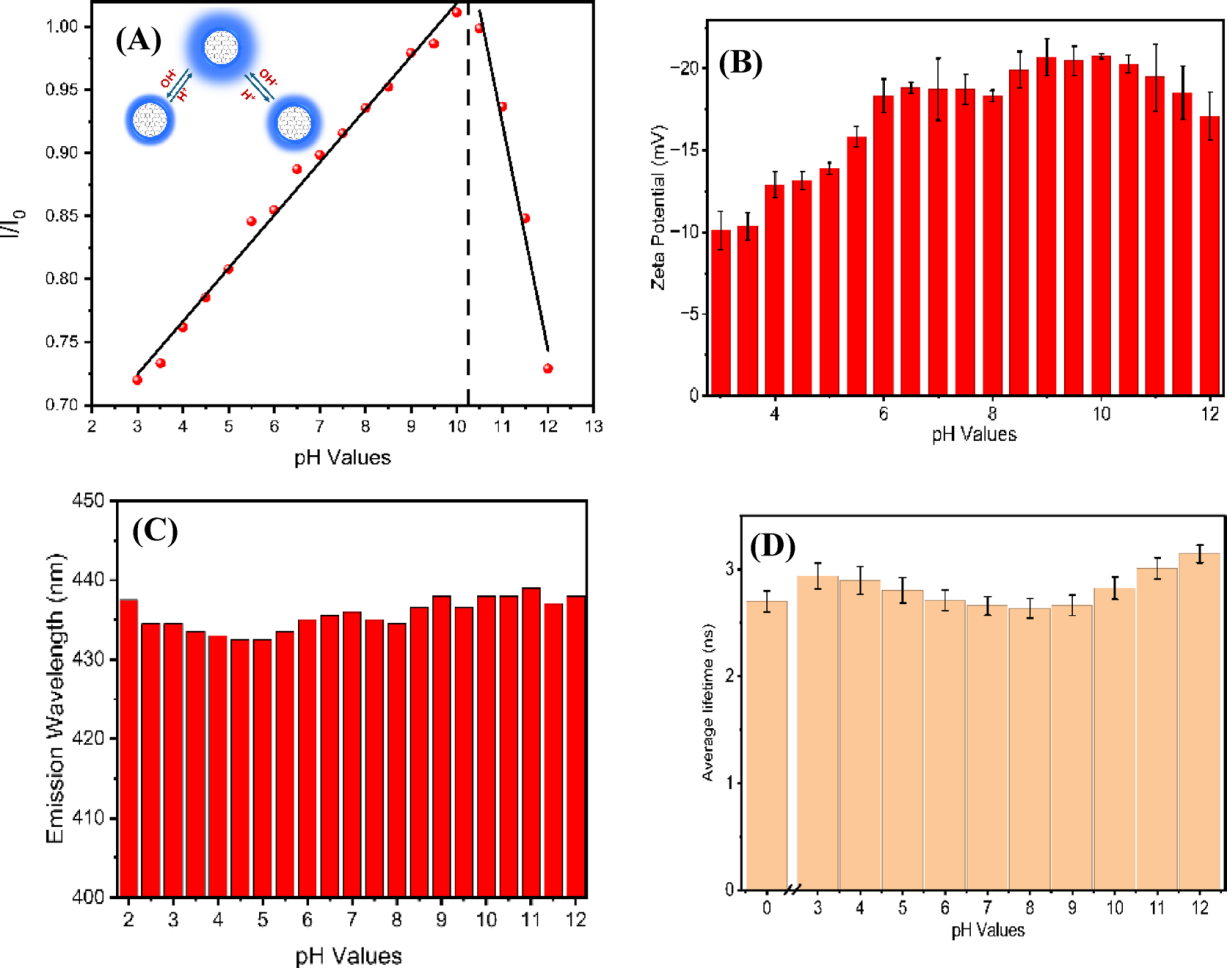
**A** The relation between PL intensity of CDs and pH ranges from 3 to 12, The inset figure shows the pH effect on the fluorescence Intensity. **B** Displays the effect of pH on the surface charge of the CD with error bars indicate the corresponding standard deviations, **C** the emission wavelength as a function of pH, demonstrating the pH-dependent fluorescence wavelength behavior, and **D** the average lifetime as a function of solution pH.

Figure 11A, illustrates that the fluorescence intensity of $\:{\mathrm{C}\mathrm{D}}_{240}^{12}$, when excited at a wavelength of 360 nm, exhibits pronounced pH-dependent behavior across a range of pH values from 3.0 to 12.0. The emission spectrum’s intensity gradually increased within the pH range of 3.0–10 and decreased in the pH range of 10.5–12.0. The inset Fig. 11A shows how the pH value affects the fluorescence intensity of CD. Additionally, two linear correlations were identified between the increases in pH value and the fluorescence intensity, represented by the equations Y=(0.599 ± 0.0071)+(0.042 ± 0.00103)x (correlation coefficient R^2^ = 0.992) and Y=(2.90 ± 0.20)−(0.179 ± 0.018)x (correlation coefficient R2 = 0.98 Across the pH range tested, our O-functionalized CDs show a monotonic increase of PL intensity from acidic to mildly alkaline conditions (≈ pH 3→10), followed by a drop at very high pH (≥ 11), while the zeta potential becomes increasingly negative with rising pH (Fig. 11B). This coupled behavior is consistent with progressive deprotonation of surface carboxyl and phenolic groups (–COOH/–OH → –COO⁻/–O⁻): deprotonation adds negative charge (driving zeta more negative) and improves colloidal stability/lowers non-radiative losses, which enhances PL up to the alkaline region; beyond ≈ pH 10–11, further deprotonation introduces new non-radiative channels, leading to PL decline. The drop in negative zeta potential is caused by a variety of variables: As the concentration of hydroxide ions increases, the surface charge is shielded by excess –OH ions, which can compress the electrical double layer around the CDs due to increased ionic strength, reducing the Zeta potential magnitude. We also thought Na + cation from added NaOH used to adjust pH would neutralize some of the surface charges. Notably, although the PL intensity changes substantially with pH, the emission maximum shifts only by a few nanometers, indicating that pH primarily modulates the population and passivation of the same emissive surface states rather than generating a new emitting species (Fig. 11C). Consistent with this interpretation, intensity–lifetime analysis shows that pH adjusts the balance between radiative and nonradiative pathways through changes in surface-state energetics: adsorption of H⁺/OH⁻ and protonation/deprotonation of oxygenated groups can shift the effective Fermi level and alter π-electron delocalization, thereby tuning HOMO/LUMO localization and the effective surface-state gap. In the pH range ~ 3–10, deprotonation generally enhances delocalization and passivates trap sites, whereas above ~ pH 10 the accumulation of negative charge can introduce additional nonradiative channels (e.g., via photoinduced electron transfer). Beyond pH ≈ 10, the accumulation of negative charge on the surface may distort the local electronic structure, promote photoinduced electron transfer from surface groups to the carbon core, all of which open additional nonradiative channels. Importantly, Fig. 11D shows that the average lifetime of the CDs is 2.9 ns at pH 3. As the pH increases, the average lifetime remains essentially unchanged, reaching only 3.1 ns at pH 12. Overall, this small variation (≈ 0.4 ns across the full pH range) indicates that pH has a negligible effect on the excited-state decay kinetics.

**Fig. 12:**
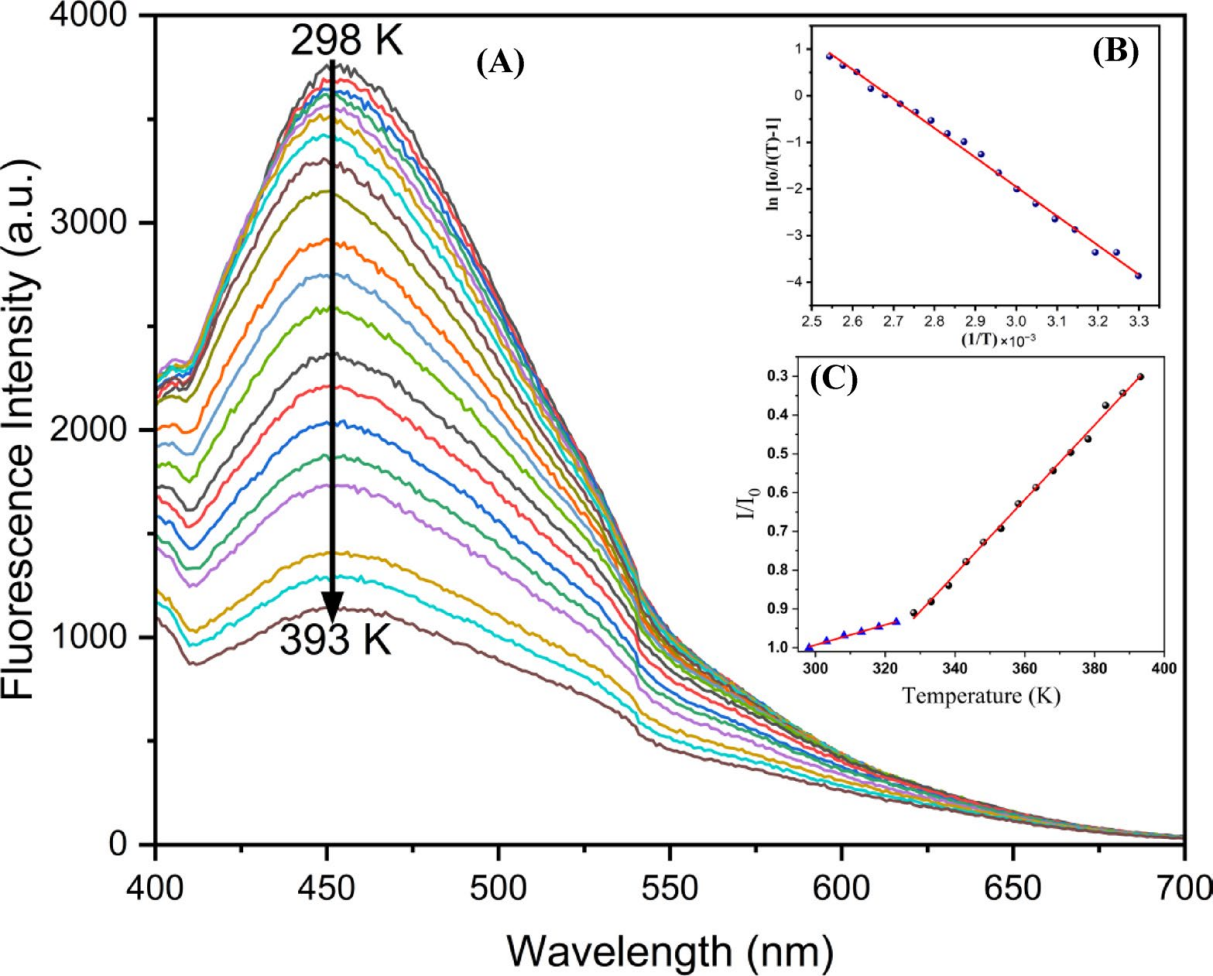
**A** PL spectra of CDs at various temperatures λ_Ex_ = 370 nm and λ_Em_ = 452 nm.; **B** The dependence of ln[(Io/I(T)−1] on 1/T. Arrhenius plot revealing the linearity of temperature-dependent emission. **C** Relative fluorescence intensity (I/I₀) as a function of temperature, highlighting the linear response.

Figure 12A shows that as the temperature increases, fluorescence decreases. The emission wavelength exhibits a negligible shift over the measured temperature range. As the temperature rises, non-radiative transitions are activated, so the PL intensity decreases^54^. Specifically, with the temperature rise, non-radiative channels are activated due to thermal effects (thermal activation). This thermal activation intensifies the non-radiative recombination of electrons and holes, which causes radiative recombination to reduce and PL to decrease. In fact, the process can be described quantitatively by using the Arrhenius equation.

$$\:\boldsymbol{I}\left(\boldsymbol{T}\right)=\frac{{\boldsymbol{I}}_{\boldsymbol{o}}}{1+\boldsymbol{C}\:\boldsymbol{e}\boldsymbol{x}\boldsymbol{p}\:(-\frac{{\boldsymbol{E}}_{\boldsymbol{a}}}{{\boldsymbol{K}}_{\boldsymbol{B}}\boldsymbol{T}})}$$

Where *E*a is the activation energy, *k* is the Boltzmann constant, and *a* is a constant. Figure 12B shows the plot of emission intensity with respect to 1/*T*, where the value of activation energy (*E*a) is calculated to be 0.542 ± 0.009 eV. Upon excitation at 370 nm, the CDs all yield an emission peak centered around 452 nm. The CDs show stability for the emission wavelength and tiny thermal broadening FWHM over the measured temperature range. However, it is found that the emission intensity decreases about 30.5% with the temperature increasing from 298 K to 393 K; intensity–temperature dependency is clearly noticeable. As the emission intensity of our CDs exhibits temperature sensitivity. In Fig. 12C, the relative PL intensity (I/I_0_) as a function of temperature can be divided into two linear regions in the temperature range from 298 K to 323 K. We can use (I/I_o_) = −0.00258 [T] + 1.767 equation to describe the linearity, from the slope of the linear fit where the R-square (R^2^) = 0.992, the relative sensitivity of this range is 0.267 K^− 1^. This trend continues in the range from 328 k to 393 k, we used the following equation to describe the linear fit: (I/I_0_) = −0.00961 [T] + 4.07925, the R-square (R^2^) = 0.997. The relative sensitivity is determined to be 1.632 K^− 1^. Compared to previous studies for using carbon dots derived from natural sources (Table 2), our carbon dots exhibit significantly enhanced thermal sensitivity. This enhancement demonstrates our synthesized CDs as an effective temperature sensor over a wide temperature range.

**Table 2:** Different natural precursors hydrothermally synthesized and used for temperature sensing, indicating their respective temperature ranges and thermal sensitivity or response.

Source	Form	Temperature Range	Thermal Sensitivity/Response	References
Coix seed	Solution phase	~ 15–80 °C	-	^55^
Castor seeds		~ 5–85 °C	Thermal sensitivity ~ 0.54% °C^–1^	^56^
Castor leaves		Not stated	Thermal sensitivity ~ 0.58% °C^–1^	^57^
Loblolly pine waste		0–60 °C	-	^58^
Eutrophic algae biomass		~ 5–44 °C	-	^59^
Cumin Seeds		~ 20–90 °C	~ 0.65% °C^− 1^	^60^
Smilax China (herbal root)		Two linear ranges: 25–40 °C and 45–80 °C	-	^61^
Aloe Vera	Solid phase	298–323 K (25̶ 50 °C)	0.267 °C^− 1^	This work
		328–393 k (55̶−20)°C	1.632 °C^− 1^	

The benchmark in Table 2, reports CDs as temperature sensors from different biomass. All studies used solution-phase CDs from natural—coix seed^55^, castor seeds/leaves^56,57^, loblolly pine waste^58^, eutrophic algae^59^, cumin seeds^60^, and Smilax china^61^—covering approximate ranges from ~ 5 to ~ 90 °C, with sensitivities reported where available (e.g., ~ 0.54–0.65%°C^–1^) and several entries laking sensitivity values. In contrast, our work features solid-phase CDs from  *Aloe Vera*, showing two linear operating regions: 298–323 K (25–50 °C) with a sensitivity of 0.267 °C^–1^ and 328–393 K (55–120 °C) with a sensitivity of 1.632 °C^–1^.

**Fig. 13:**
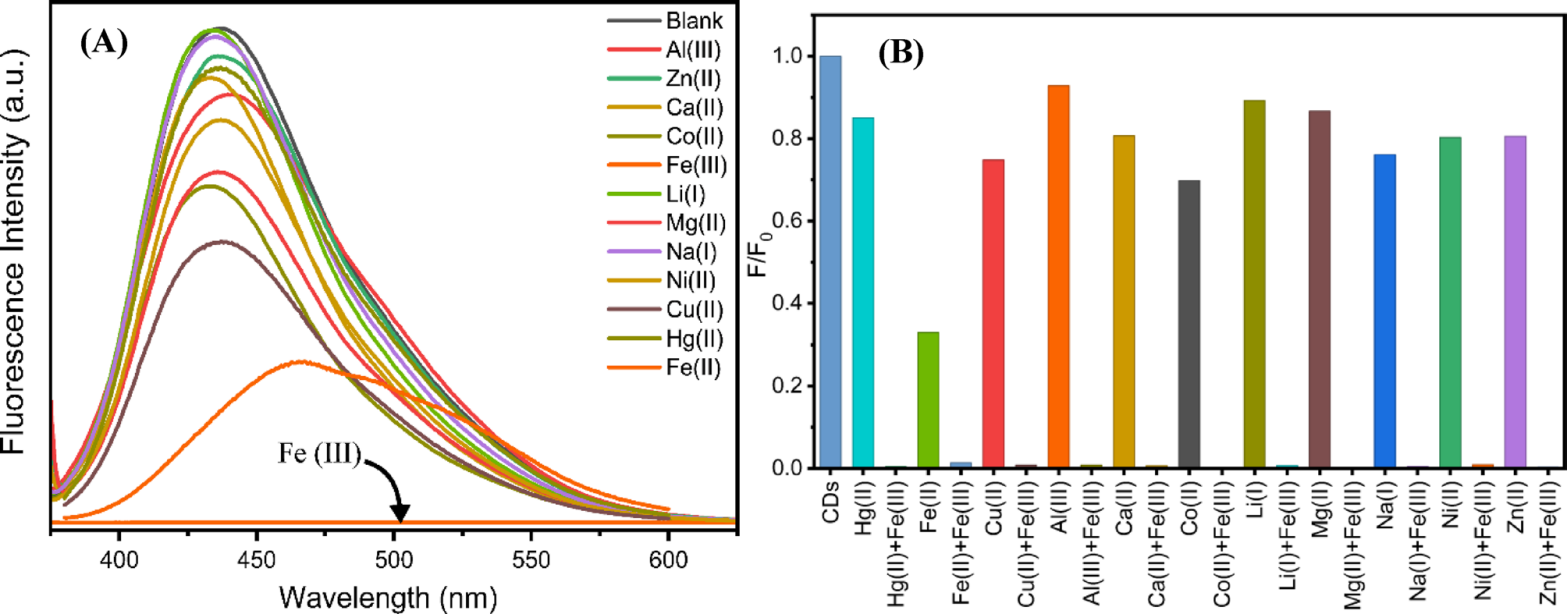
**A** The effect of addition of different metal ions on PL intensity. **B** Interference experiment was performed by measuring the fluorescence response (F0/F) of CQDs to Fe (III) in the presence of various metal ions.

To investigate the potential of the *Aloe vera* CDs as a sensor for metal ions, CDs synthesized at 180 °C for 4 h and at 240 °C for 4 and 12 h were used as FL sensor metal ions detection. CDs solutions were mixed with 0.1 mM concentration of different metal ions: Al (III), Ca (II), Co (II), Fe (III), Li (I), Mg (II), Na (I), Ni (II), Zn (II), Cu (II), Hg (II), and Fe (II). PL intensity of CDs was measured at excitation wavelength 360 nm at room temperature. All samples show similar results when metal ions are added to the solutions. The intensity spectra were plotted Among all tested metal ions, Fe^3+^ exhibited the most pronounced quenching effect, resulting in PL intensity of the CDs solution to nearly zero (Fig. 13A). Additionally, a quantitative interference experiment was conducted to further evaluate the interaction of CDs with Fe^3+^ ions and the presence of other metal ions. Figure 13B illustrates that the fluorescence intensity of the CDs in mixed-ion solutions was significantly quenched only in the presence of Fe^3+^, whereas the effect of other metal ions was not significant. This finding indicates that the CDs show a significant affinity and notable selectivity for Fe^3+^, even in the presence of several opposing factors. This significant quenching indicates a strong interaction between Fe (III) ions and oxygen functional groups present on the CDs, leading to complex formation that alters the emission spectra. Therefore, *Aloe vera* CDs exhibit high selectivity and sensitivity as efficient sensors for Fe (III) ions.

**Fig. 14:**
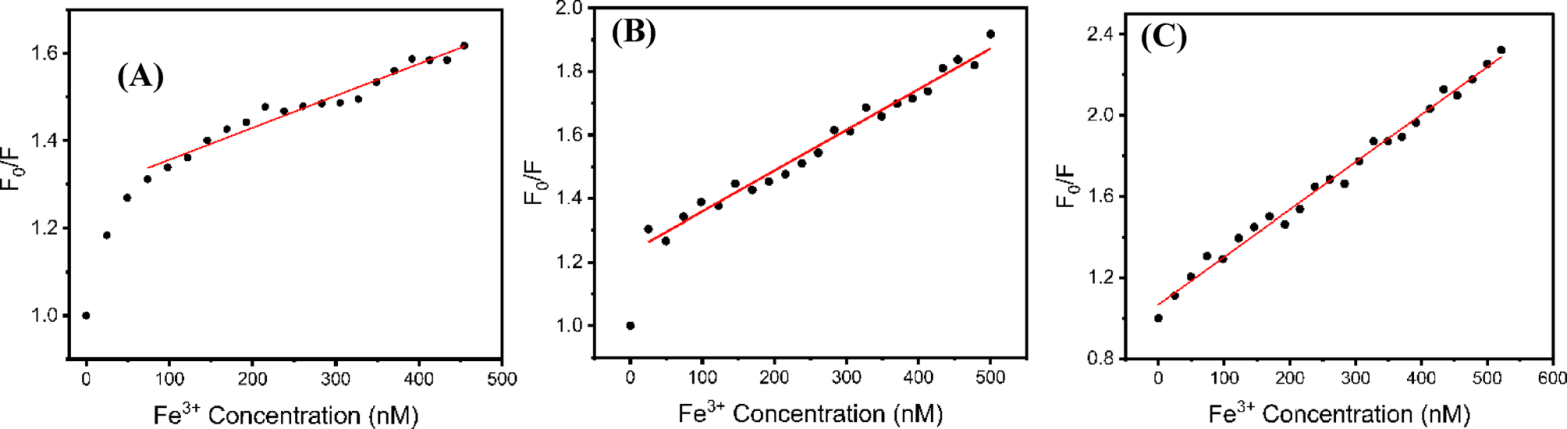
The linear calibration range for F_0_/F and Fe^3+^ Concentration for $\:{CD}_{180}^{4}$ (**A**), $\:{CD}_{240}^{4}$ (**B**), and ${CD}_{240}^{12}$ (**C**). Demonstrating that higher synthesis temperature and longer reaction times significantly enhance the sensitivity of the CDs toward Fe³⁺ detection.

Sensitivity tests were performed for $\:{CD}_{180}^{4}$, $\:{CD}_{240}^{4}$, and $\:{CD}_{240}^{12}$, and different concentrations of Fe^3+^ in range 0- 500 nmol L^− 1^ were added to the CDs solution, and the fluorescence intensities were recorded under 360 nm excitation (Fig. 14). The observation demonstrates that the fluorescence intensity steadily decreases with an increase in Fe³⁺ concentration that fluorescence intensities follow the stern-Volmer equation:

$$\:\frac{{F}_{0}}{F}-1={K}_{sv}\left[C\right]$$

where F_0_ and F are the emission intensities of the CDs at 360 nm in the absence and presence of Fe^3+^, respectively; K_sv_ is the Stern–Volmer burst constant; and [C] is the Fe^3+^ concentration.

**Fig. 15:**
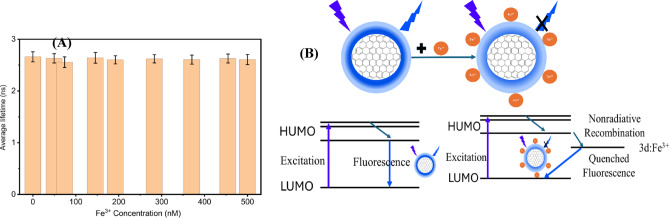
**A** The average lifetime of CD as a function of Fe³⁺ concentration (0–500 nM). **B** Diagram illustrating how CD interacts with Fe³⁺ ions.

Table 3 summarizes the key parameters of the synthesized CD samples. The limit of detection (LOD) was calculated using the Eq. 3σ/k, where k represents the slope obtained from the linear fit and σ denotes the standard deviation of the intercept. Adjusting the synthesis parameters markedly enhanced the detection sensitivity, improving the LOD from 43.78 nM to 16.15 nM. Upon addition of 500 nM Fe(III), the fluorescence intensity dropped markedly, corresponding to a quenching efficiency of 55.62%. Table 3 compares the LOD values obtained in this work with those reported in previous studies and indicates that CDs prepared from *Aloe vera* are efficient candidates for the detection of Fe³⁺ ions. Figure 15A shows the $\:{CD}_{240\:}^{12}$average lifetime study as function of Fe³⁺ concentration. The nearly unchanged lifetime indicates that Fe³⁺ induced fluorescence quenching is dominated by a static interaction rather than dynamic quenching. The quenching of fluorescence in CD by Fe^3+^ is mainly due to the strong interactions between Fe^3+^ ions and the oxygen-containing surface groups that exist on the carbon dots, especially the –OH functionalities. The groups exhibit the ability to chelate Fe^3+^ via inner-sphere coordination, offering multiple oxygen donor sites and a polar environment that leads to complexation. The high density of oxygen donor sites on the CD surface explains the significant selectivity for Fe^3+^ (Fig. 15B). Consequently, stable non-emissive CD–Fe^3+^ complexes are generated, resulting in fluorescence quenching through a static mechanism. In this scenario, the number of emissive species diminishes, yet the excited-state lifetime of the remaining fluorophores stays constant.

**Table 3:** Calibration parameters for the cds samples as a sensor of Fe³+.

Parameters	$\:{CD}_{180}^{4}$	$\:{CD}_{240}^{4}$	$\:{CD}_{240}^{12}$
Linear Range (nM)	74.89̵ 454.55	24.87 -500	0 -500
K_sv_ (M^− 1^)	0.733 $\:\times\:10$^6^	1.28 $\:\times\:10$^6^	2.32 $\:\times\:10$^6^
LOD (nM)	43.78	28.8	16.15
R^2^	0.96	0.98	0.995

In Table 4, hydrothermally prepared CDs from different biomass precursors show LODs with wide ranges from the tens-of-nanomolar to the low-micromolar level. Our CDs achieved the best LOD (16.15 nM), compared to lychee waste (23.6 nM)^62^, potatoes (25 nM)^63^, and candle soot (40 nM)^64^. Other different sources such as cranberry beans (9.55 µM)^65^ and B. flabellifer (2.01 µM)^66^ exhibit higher LODs. The linear dynamic ranges also vary widely: several systems afford broad ranges (e.g., water hyacinth, 0–330 µM^62^; cranberry beans, 30–600 µM^65^; glycine, 0.5 µM–0.5 mM)^67^. Among all precursors, *Aloe Vera* CDs achieved the lowest LOD of 16.15 nM. Its working range (0–0.5 µM) is narrower than potatoes (1–5 µM) and candle soot, 0.16–2.0 µM), reflecting a design optimized for ultra-low concentrations.

**Table 4:** Comparison of the linear detection ranges and limits of detection (LOD) for different carbon Dot (CD) sources for Fe^3+^ ion sensing applications.

Carbon Dot Source	Synthesis Method	LOD	Linear Range	Reference
Barberry (Berberis sp. fruit)	Hydrothermal	1.73 µM​	5–80 µM	^68^
Borassus flabellifer (ice apple)	Hydrothermal	2.01 µM​	10–100 µM	^66^
Lychee fruit waste	Solvothermal ​	23.6 nM​	0–22 µM	​^62^
Water hyacinth	Hydrothermal	84 nM	0–330 µM	^69^
Cranberry beans	Hydrothermal ​	9.55 µM​	30–600 µM	^65^​
Mint leaves (Mentha)	Hydrothermal ​	0.374 µM	0–0.38 µM	^70^
Rose-heart radish	Hydrothermal	0.13 µM	0.02–40 µM	^71^
Onion waste	Hydrothermal	0.21 µM	0–20 µM	^72^
Bombyx mori silk	Hydrothermal	0.38 µM	0.5˗4.0 µM	^73^
Glycine	Hydrothermal	0.1 µM	0.5 µM − 0.5 mM	^67^
Coriander leaves	Hydrothermal	0.4 µM	0–6.0 µmol/L^–1^	^74^
Potatoes	Hydrothermal	25 nM	1.0–5.0 µM	^63^
Candle soot	Chemical oxidation/reflux	40 nM	0.16–2.0 µM	^64^
*Aloe vera*	Hydrothermal	16.15 nM	0–0.5.5 µM	This work

To validate the practical applicability of the CD-based sensor for Fe³⁺ detection, a tap-water sample was collected from the elemental analysis laboratory at the Egypt–Japan University of Science and Technology. The sample was filtered through a 45 μm syringe filter to remove suspended impurities that might interfere with sensor performance. Using the established protocol, aliquots of 20, 40, 60, 80, and 100 µL of a 5 mM Fe³⁺ solution were added to a mixture of 1 mL CDs and 0.5 mL tap water. The corresponding Fe³⁺ concentrations were calculated via the dilution equation and compared with values obtained from the calibration curve (Table 5). Recovery values ranged from 90.71% to 106.09%, and the relative standard deviation for triplicate measurements remained below 5%. These analytical results show that our CDs-based sensor is effective at detecting Fe³⁺ in tap water.

**Table 5:** Detection Fe^3+^ ion in tap water and RSD (*N* = 3).

Sample	Fe^3+^ Added (µM)	Fe^3+^Founded (µM)	Recovery (%)	RSD (%)
	65.8	61.99	94.22	2.36
Tap water	130	137.92	106.09	0.21
	192	178.89	93.18	0.55
	253	229.49	90.71	3.86
	312.5	327	104.64	1.68

## Conclusion

Carbon dots (CDs) were synthesized from *Aloe vera* gel using a green, one-step hydrothermal route, and the combined influence of reaction temperature (180–240 °C) and time (4–16 h) was systematically mapped across 16 synthesis conditions. This design provides more than “best-condition reporting”: it establishes a synthesis–structure–property relationship for biomass-derived CDs by correlating changes in morphology/crystallinity and oxygenated surface chemistry with optical response. The optimized condition (240 °C, 12 h) delivered the highest PLQY 3.5%) and was therefore selected as a rationally optimized fluorescent platform rather than an empirically chosen sample. A key novelty of this work is demonstrating Aloe vera CDs as a solid/powder-state fluorescent temperature probe operating from 298 to 393 K, with two linear working regions and enhanced relative sensitivities (0.267 K^–1^ at 298–323 K and 1.632 K^–1^ at 328–393 K). In addition, the same CDs function as a wide-range pH probe (pH 3–12) with two calibration regimes, enabling quantitative readout across acidic to strongly alkaline conditions. For metal-ion sensing, the CDs show pronounced selectivity toward Fe³⁺ and achieve an ultra-low LOD of 16.15 nM (linear range: 0–500 nM), which is reported in this study as the lowest among hydrothermally synthesized CDs from natural precursors; lifetime behavior supports a predominantly static quenching mechanism consistent with complex formation at oxygen-rich function groups. Beyond sensitivity, the practical importance is supported by real-sample applicability: fluorescence remains nearly constant under high ionic strength (NaCl up to 666 mM), and only gradual photodegradation is observed during 360 nm irradiation. Finally, Fe³⁺ detection in tap water produced acceptable recoveries (90.71–106.09%) with RSD < 5%, demonstrating feasibility for environmental/water-monitoring workflows using a low-cost, sustainable precursor. Overall, this investigation advances  *Aloe Vera*–derived CDs from single-function fluorescent materials toward a multifunctional, application-ready platform, while also providing a transferable optimization strategy for engineering biomass-derived CDs with purpose-oriented sensing performance.

## Supplementary Information

Below is the link to the electronic supplementary material.

Supplementary Material 1

## Data Availability

Data availabilityAll data generated or analyzed during this study are included in this published article.
