# Supplementary material for: Aloe vera derived carbon dots as multifunctional fluorescent probe for temperature, pH, and ferric ion sensing
Source: Sci Rep. 2026 Jan 20;16:2600. doi: 10.1038/s41598-025-34499-x (PMC12820301; doi:10.1038/s41598-025-34499-x)
Supplement: Supplementary file 1 — Supplementary Material 1 [file 41598_2025_34499_MOESM1_ESM.docx]

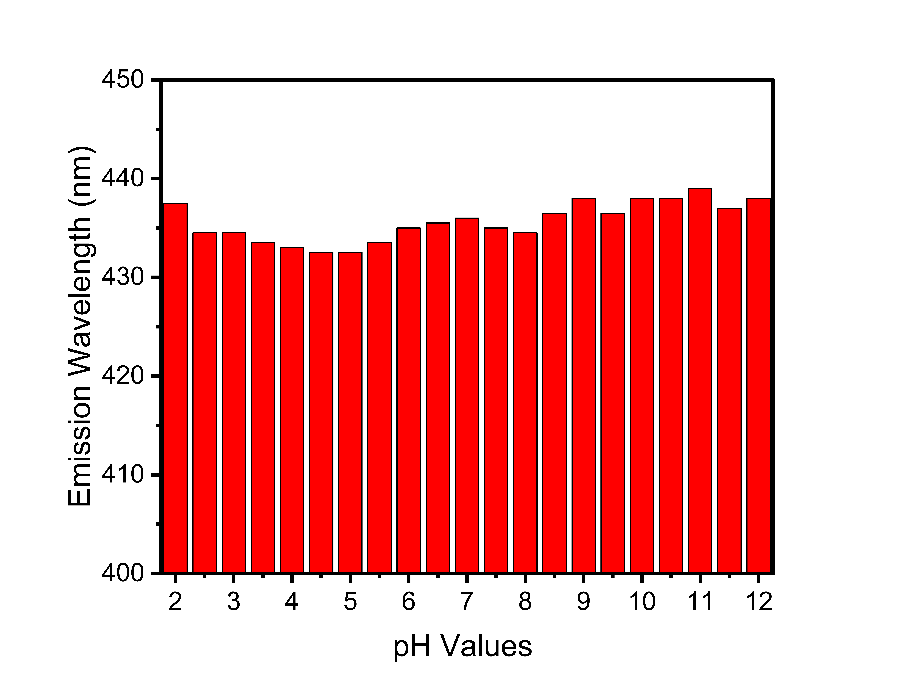


Figure 1: the emission wavelength as a function of pH, demonstrating the pH-dependent fluorescence wavelength behavior.


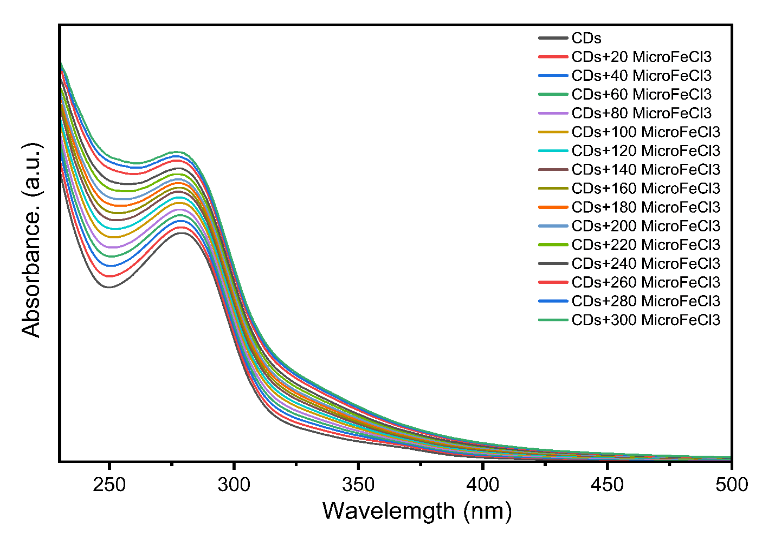


Figure 2: UV–vis absorption spectra of carbon dots with different FeCl₃ volumes.
